# Minocycline promotes the generation of dendritic cells with regulatory properties

**DOI:** 10.18632/oncotarget.10810

**Published:** 2016-07-24

**Authors:** Narae Kim, Chan-Su Park, Sun-A Im, Ji-Wan Kim, Jae-Hee Lee, Young-Jun Park, Sukgil Song, Chong-Kil Lee

**Affiliations:** ^1^ College of Pharmacy, Chungbuk National University, Cheongju, South Korea

**Keywords:** minocycline, dendritic cell, growth promotion, regulatory property, CD4 Tregs, Immunology and Microbiology Section, Immune response, Immunity

## Abstract

Minocycline, which has long been used as a broad-spectrum antibiotic, also exhibits non-antibiotic properties such as inhibition of inflammation and angiogenesis. In this study, we show that minocycline significantly enhances the generation of dendritic cells (DCs) from mouse bone marrow (BM) cells when used together with GM-CSF and IL-4. DCs generated from BM cells in the presence of minocycline (Mino-DCs) demonstrate the characteristics of regulatory DCs. Compared with control DCs, Mino-DCs are resistant to subsequent maturation stimuli, impaired in MHC class II-restricted exogenous Ag presentation, and show decreased cytokine secretion. Mino-DCs also show decreased ability to prime allogeneic-specific T cells, while increasing the expansion of CD4^+^CD25^+^Foxp3^+^ T regulatory cells both *in vitro* and *in vivo*. In addition, pretreatment with MOG35-55 peptide-pulsed Mino-DCs ameliorates clinical signs of experimental autoimmune encephalitis induced by MOG peptide injection. Our study identifies minocycline as a new pharmacological agent that could be potentially used to increase the production of regulatory DCs for cell therapy to treat autoimmune disorders, allergy, and transplant rejection.

## INTRODUCTION

Dendritic cells (DCs) are professional APCs with a unique role in the generation of T cell-mediated immune responses and maintenance of immunological tolerance [1-3]. DC ability to guide T cell responses depends on their state of maturation and functional differentiation, which is influenced by environmental factors such as microbial products, cytokines, cyclooxygenase metabolites, and immunosuppressive drugs [4, 5]. For instance, DC exposure to an immunosuppressive drug rapamycin triggers the development of tolerogenic DC population [6]. Tolerogenic DCs are characterized by reduced expression of costimulatory molecules and IL-12, and decreased ability to induce T cell proliferation. At the same time, tolerogenic DCs produce increased levels of IL-10 and are potent stimulators of regulatory T cells (Tregs) [6-8] by generating inhibitory signals through increased expression of Programmed death-ligand 1 (PD-L1) and Inducible T-cell co-stimulator (ICOS) [9, 10].

Pharmacological agents known to induce regulatory DCs include vitamin D3, dexamethasone, rapamycin, corticosteroid, aspirin, atorvastatin, retinoic acid, and mycophenolic acid [7]. Immunosuppressive cytokines such as IL-10 and TGF-β have also been shown to induce regulatory DCs [11]. Regulatory DCs generated with IL-10 [12-14], vitamin D3 [15-17], dexamethasone [18-20], and rapamycin [6, 21-23] have been studied in experimental animals and in humans with the aim to develop clinical approaches for the prevention of transplantation rejection and treatment of autoimmune and chronic inflammatory conditions. In recent years, clinical studies have been launched to evaluate the ability of human tolerogenic DCs to suppress autoimmunity [24-26].

Tetracyclines are a group of antibiotics that have been widely used to treat infectious diseases for over 50 years. Recently, these broad-spectrum antimicrobial agents have been confirmed to exhibit a number of non-antibiotic properties such as anti-inflammatory, immunomodulatory, and neuroprotective activities [27]. Over 100 clinical trials have been performed to investigate the potential of tetracyclines in the treatment of immune system diseases [27, 28]. Minocycline (7-dimethylamino-6-dimethyl-6-deoxytetracycline) is a second-generation semi-synthetic tetracycline with over 30 years of clinical use as an antibiotic. Similar to other tetracyclines, minocycline has recently been shown to exert various non-antibiotic effects, including inhibition of microglia proliferation and the release of inflammatory mediators such as NO, TNF-α, IL-1β, and IL-6 [29]. Minocycline has already been adopted in the treatment of autoimmune disorders such as rheumatoid arthritis, inflammatory bowel diseases, scleroderma, aortic aneurysms, and periodontitis [30-32].

In this study, we show that minocycline significantly increased the generation of DCs from mouse bone marrow (BM) cells when used together with GM-CSF and IL-4. The minocycline-conditioned DCs (Mino-DCs) had reduced APC function, did not mature in response to LPS or cytokine stimulation, and were poor allogeneic stimulators both *in vitro* and *in vivo*. To further examine minocycline activity *in vivo*, it was used together with GM-CSF to generate splenic Mino-DCs in mice. Splenic Mino-DCs were also severely impaired in priming alloantigen-specific T cells in allogeneic mice, and increase CD4^+^CD25^+^Foxp3^+^ T regulatory cells. To the best of our knowledge, this is the first report of the application of minocycline to increase the production of regulatory DCs.

## RESULTS

### Minocycline increases the generation of BM-derived DCs

**Figure 1:**
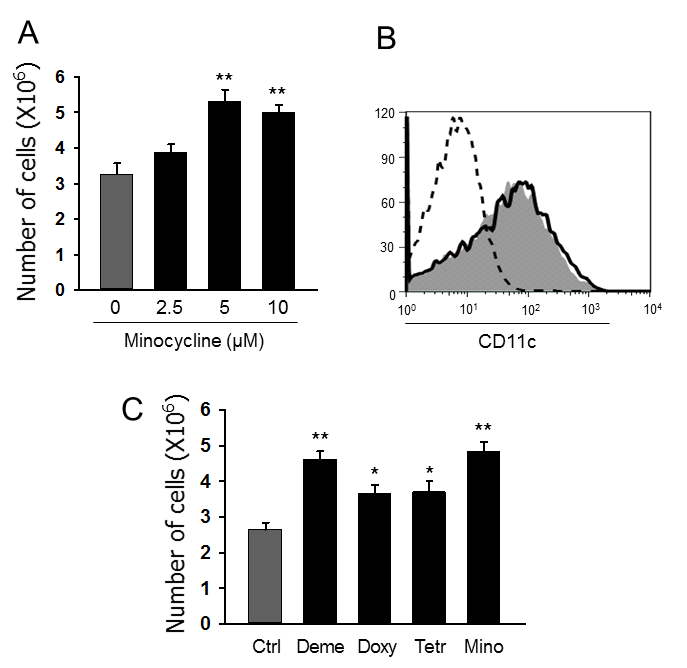
Minocycline enhances the generation of DCs from BM cells **A.** DCs were generated from C57BL/6 mouse BM cells using GM-CSF (40 ng/ml) and IL-4 (40 ng/mL) without (Ctrl-DCs) or with indicated concentrations of minocycline (Mino-DCs) for 7 days. The data are presented as the mean ± SD of six independent experiments; **P < 0.01. **B.** Representative histograms showing CD11c expression in DCs generated with 5 μM minocycline. Solid line, Ctrl-DCs; shaded area, Mino-DCs; dotted line, isotype-matched control. **C.** DCs were generated from C57BL/6 mouse BM cells using GM-CSF and IL-4 with indicated concentrations of tetracycline antibiotics for 7 days (Deme: demeclocycline; Doxy: doxycycline; Tetr: tetracycline; Mino: minocycline). The data are presented as the mean ± SD of six independent experiments; * *P* < 0.05, **P < 0.01 compared with control.

Myeloid DCs were generated from mouse BM cells after 7-day culture with GM-CSF plus IL-4 (40 ng/ml each) in the presence (Mino-DCs) or absence (Ctrl-DCs) of different concentrations of minocycline. The addition of minocycline significantly and dose-dependently increased the total number of DCs in culture (Figure 1A). While an average of 3.3 × 10^6^ Ctrl-DCs were obtained from 6.0 × 10^6^ plated BM cells, the exposure to 5 μM minocycline resulted in the generation of 5.3 × 10^6^ Mino-DCs, which indicated a 61% increase. Flow cytometry analysis revealed that Mino-DCs and Ctrl-DCs expressed similar levels of surface CD11c (Figure 1B).

We then examined whether other tetracyclines also exert DC generation-enhancing activity. At 5 μM concentration, all the tetracycline derivatives tested including demeclocycline, doxycycline and tetracycline were significant enhancers of DC generation from BM cells (Figure 1C). Among them, minocycline demonstrated the highest activity and was chosen for further experiments. In subsequent experiments, we generated Mino-DCs using 5 μM minocycline, 40 ng/ml GM-CSF, and 40 ng/ml IL-4.

### Mino-DCs are refractory to maturation

**Figure 2:**
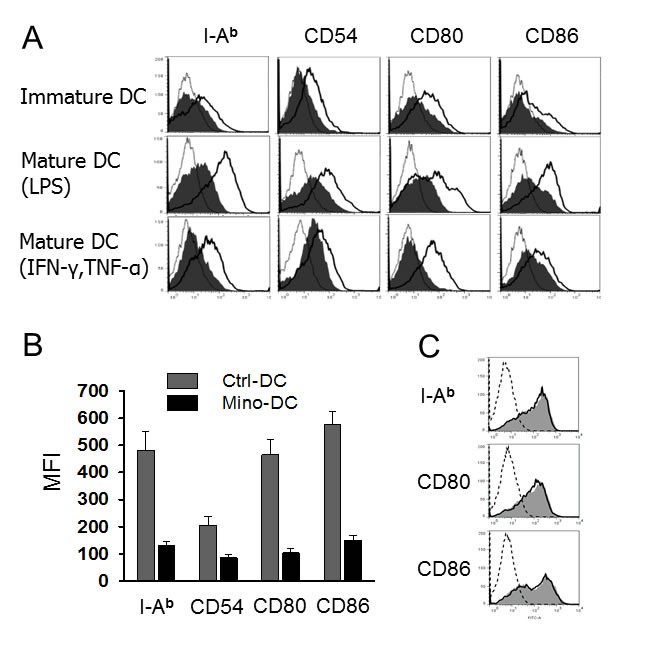
DCs generated in the presence of minocycline resist maturation **A.** Immature DCs generated from C57BL/6 mouse BM cells with (Mino-DCs) or without (Ctrl-DCs) minocycline were exposed to 100 ng/ml LPS or 50 ng/mL IFN-γ plus 50 ng/mL TNF-α for 24 h to induce maturation and analyzed by flow cytometry for the expression of I-A^b^, CD54, CD80 and CD86. Mino-DCs, shaded area; Ctrl-DCs, black line; isotype-matched control, grey line. **B.** Mean fluorescence intensities of LPS-stimulated DCs. The data are presented as the mean ± SD of three independent experiments. **C.** Immature Ctrl-DCs were stimulated with 50 ng/mL IFN-γ and 50 ng/mL TNF-α in the presence (shaded area) or absence (solid lines) of minocycline for 24 h and analyzed by flow cytometry; dotted line, isotype-matched control.

Immature Mino-DCs expressed slightly lower levels of MHC class II (I-A^b^), CD54, CD80, and CD86 compared to immature Ctrl-DCs (Figure 2A). Both cell populations were induced to maturation by exposure to 100 ng/ml LPS or 50 ng/mL IFN-γ plus 50 ng/mL TNF-α for 24 h. Mino-DCs were obviously refractory to maturation as evidenced by significantly lower expression of mature DС surface markers compared to Ctrl-DCs (Figure 2A, 2B). It is noteworthy that, for Mino-DC generation, minocycline was added to BM cells from the initiation of culture. To examine the effects of minocycline on DC maturation, immature DCs were generated with GM-CSF and IL-4 in the absence of minocycline, and then stimulated with LPS for 24 h in the presence of minocycline. Addition of minocycline to normal immature DCs did not impair LPS-induced phenotypic maturation (Figure 2C).

### Mino-DCs have reduced cytokine secretion ability

**Figure 3:**
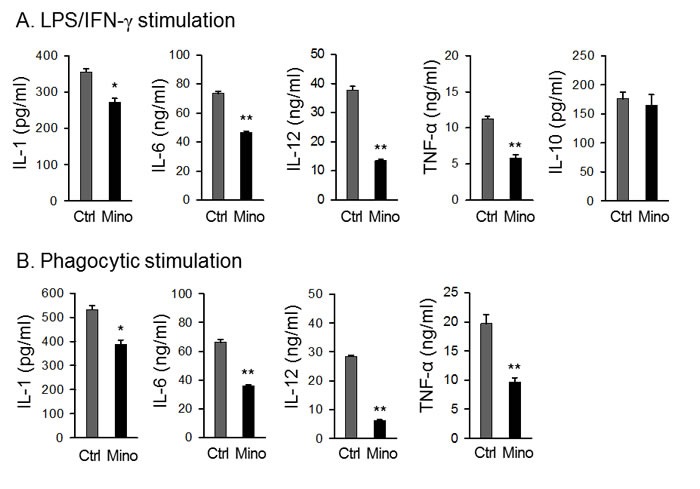
DCs generated in the presence of minocycline are deficient in cytokine secretion Immature DCs generated from C57BL/6 mouse BM cells with (Mino-DCs) or without (Ctrl-DCs) minocycline were stimulated with 100 ng/ml LPS (A) or biodegradable OVA-microspheres (50 μg/ml as OVA) (B) for 24 h. For induction of IL-10 production, DCs were stimulated with 100 ng/ml LPS and 50 ng/mL IFN-γ. Cytokine secretion to culture supernatant was determined by ELISA. The data are presented as the mean ± SD of three separate experiments; * *P* < 0.05, **P < 0.01 compared with control.

Mino-DCs produced significantly lower levels of proinflammatory cytokines IL-12, IL-1, IL-6, and TNF-α in response to LPS stimulation compared to Ctrl-DCs (Figure 3A). However, there was no difference in IL-10-producing capability between Mino-DCs and Ctrl-DCs (Figure 3A). Phagocytosis by itself could be an activation signal to DCs. When biocompatible/biodegradable OVA-microspheres were used as particles to induce phagocytic activation, Mino-DCs still secreted much lower amounts of proinflammatory cytokines compared to Ctrl-DCs (Figure 3B). Phagocytic stimulation of the DCs with OVA-microspheres was unable to induce IL-10 production.

### Mino-DCs are impaired in MHC class II-restricted exogenous Ag presentation

**Figure 4:**
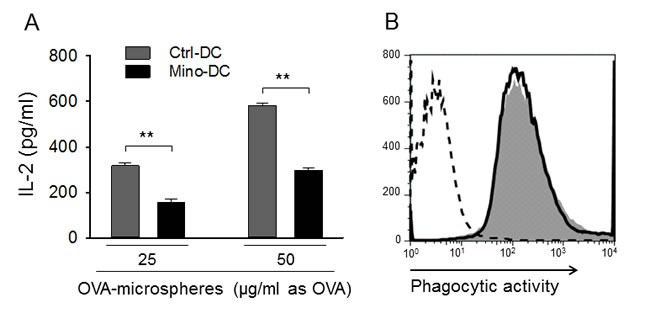
DCs generated in the presence of minocycline are impaired in Ag presentation **A.** Immature DCs generated from BALB/c mouse BM cells with (Mino-DCs) or without (Ctrl-DCs) minocycline were incubated with OVA-microspheres (25 or 50 μg/ml as OVA) for 1 h; not phagocytosed OVA-microspheres were removed and cells were fixed and co-cultured with DOBW cells which recognize OVA [323-339]-I-A^d^ complexes. IL-2 secretion by DOBW cells was measured by ELISA. The data are presented as the mean ± SD of five independent experiments; **P < 0.01. **B.** DCs were incubated with FITC-labeled OVA-microspheres for 2 h and analyzed by flow cytometry. Shaded area, Mino-DCs; solid line, Ctrl-DCs; dotted line, DCs not incubated with OVA-microspheres.

APC functions of generated DCs were determined after phagocytosis of OVA-microspheres. After 2-h incubation with microspheres, cells were fixed and analyzed for the presentation of surface OVA peptide-class II MHC complexes by measuring IL-2 production by OVA-specific CD4 T cell hybridoma DOBW cells. Mino-DCs were found inefficient in MHC class II-restricted exogenous Ag presentation compared with Ctrl-DCs as evidenced by reduced IL-2 levels secreted by CD4 T cell (Figure 4A). However, the difference was not due to the deficiency in phagocytic activity of Mino-DCs, which was almost similar for both cell populations (Figure 4B). The suppressed APC function may be due to the decreased expression of MHC class II and co-stimulatory molecules on the cell surface (Figure 2).

### Mino-DCs are deficient in allogeneic T cell stimulation both *in vitro* and *in vivo*

**Figure 5:**
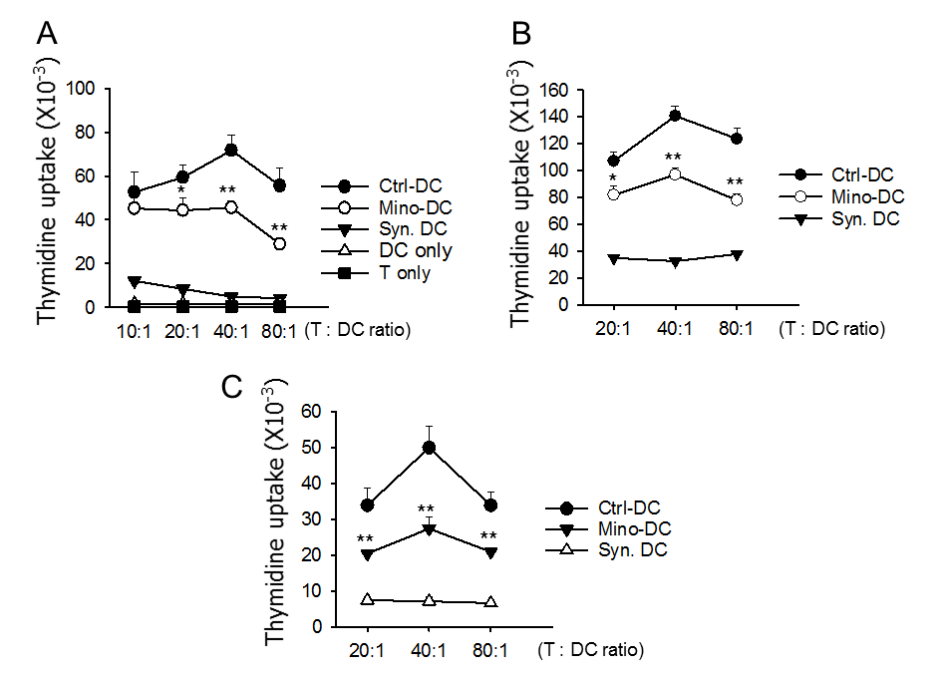
DCs generated in the presence of minocycline are deficient in allogeneic T cell priming **A.** Immature DCs generated from C57BL/6 mouse BM cells with (Mino-DCs) or without (Ctrl-DCs) minocycline were stimulated with LPS for 24 h for maturation, and then co-cultured for three days with CD4^+^ T cells isolated from the spleens of BALB/c mice at the indicated ratios. DNA synthesis was measured by the incorporation [^3^H]-thymidine added for the final 18 h of culture. **B.** Mino-DCs and Ctrl-DCs generated *in vitro* were transferred to BALB/c mice (1 × 10^6^/mouse). Ten days later, T cells were isolated from the spleens and re-stimulated with normal (C57BL/6 mouse BM-generated) DCs at the indicated ratios. DNA synthesis was measured as above. DCs generated from BALB/c mouse BM ells served as a syngeneic control (Syn-DC). The data are presented as the mean ± SD of three independent experiments. **C.** C57BL/6 mice were injected i.p. with GM-CSF and minocycline (Mino-DC) or GM-CSF (Ctrl-DC) for 6 days. On day-7, CD11c^+^ cells were isolated from the spleens and transferred to BALB/c mice (1 × 10^6^/mouse). DCs generated from BALB/c mouse BM cells served as a syngeneic control (Syn-DC). After 10 days, total T cells were isolated from the spleens and co-cultured with normal C57BL/6 BM-derived DCs. DNA synthesis was measured as above. The data are presented as the mean ± SD of three independent experiments; *P < 0.05, **P < 0.01 compared with the Ctrl-DC group.

The ability of Mino-DCs to prime allogeneic T cells was analyzed using the mixed lymphocyte reaction assay. In this experiment, Ctrl-DCs and Mino-DCs generated from BM cells of C57BL/6 mice were induced to maturation with LPS, and then co-cultured with CD4^+^ T cells isolated from BALB/c mice. Ctrl-DCs were efficient in inducing allogeneic T cell proliferation; maximum stimulatory activity was observed at the T cell to DC ratio of 40:1. However, allostimulatory activity of Mino-DCs was significantly lower than that of Ctrl-DCs as evidenced by reduced [^3^H]-thymidine uptake by CD4^+^ T cells (Figure 5A).

The ability of Mino-DCs to prime allogeneic T cells was also examined in allogeneic mice. In this experiment, LPS-stimulated Mino-DCs derived from C57BL/6 mice were injected into BALB/c mice. Seven days later, T cells were isolated from the spleens and re-stimulated with normal DCs generated from BM cells of C57BL/6 mice. The results indicated that Mino-DCs had a significantly reduced activity to prime alloantigen-specific T cells in allogeneic mice (Figure 5B).

To bolster that minocycline induces DCs with low allostimulatory capacity *in vivo*, Mino-DCs were generated in mice that were injected with minocycline. In this experiment, C57BL/6 mice received GM-CSF and minocycline for 6 days, while control mice received GM-CSF alone. On day-7, spleen CD11c^+^ cells were isolated from each group, and were used to immunize BALB/c mice. After 10 days, total spleen T cells were isolated and co-cultured with normal C57BL/6 BM-derived DCs. As shown in Figure 5C, immunization of BALB/c mice with Ctrl-DCs strongly induced alloantigen-specific T cell responses in BALB/c mice. In contrast, Mino-DCs isolated from mice treated with GM-CSF plus minocycline exhibited significantly weaker allopriming capacity in BALB/c mice. Consistent with these results, Mino-DCs isolated from the spleens of mice treated with GM-CSF plus minocycline expressed significantly lower levels of I-A^b^ and CD80 compared to Ctrl-DCs isolated from the spleens of mice treated with GM-CSF alone (Supplementary Figure 1).

### Mino-DCs induce Foxp3^+^ Treg cells

**Figure 6:**
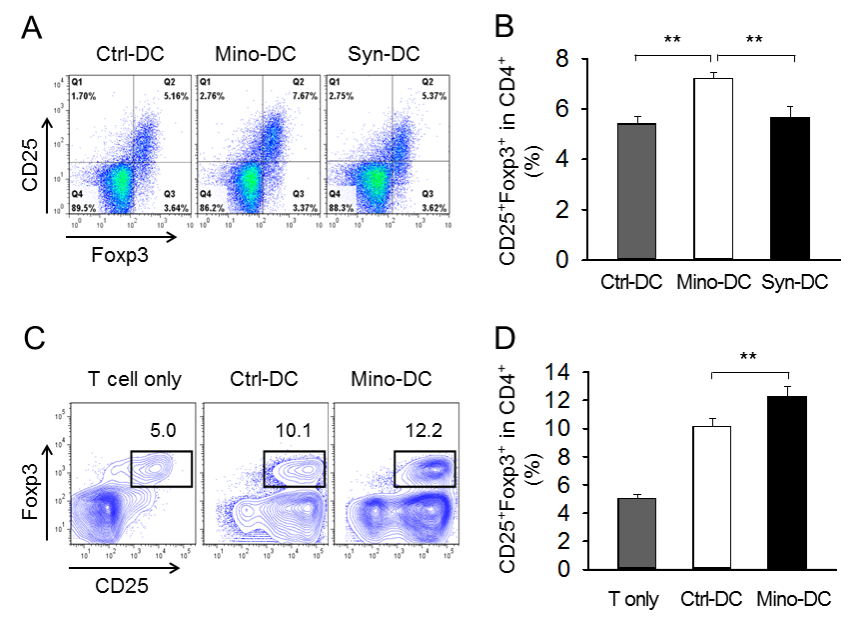
DCs generated in the presence of minocycline induce Foxp3^+^ Treg cells **A.** Immature DCs generated from C57BL/6 mouse BM cells with (Mino-DCs) or without (Ctrl-DCs) minocycline were stimulated with LPS for 24 h to mature, and then transferred to BALB/c mice (1 × 10^6^/mouse). DCs generated from BM cells of BALB/c mice served as a syngeneic DC control. After 7 days, spleen cells were isolated and subjected to flow cytometry. Cells were gated on CD4^+^ cells, and analyzed for the expression of CD25 and Foxp3. **B.** The proportion of CD25^+^Foxp3^+^ T cells in the CD4^+^ cell population of each experimental group. **P < 0.01. **C.** Immature DCs generated from C57BL/6 mouse BM cells with (Mino-DCs) or without (Ctrl-DCs) minocycline were stimulated to maturity with IFN-γ plus TNF-α for 24 h, pulsed with the OVA323-339 peptide (10 μg/ml), washed, and co-cultured with CD4^+^ T cells isolated from OT-II mice in the presence of 100 U/ml IL-2. After 4 days, cells were harvested and stained as described in (A). Cell populations were gated on CD4^+^ cells, and then analyzed for the expression of CD25 and Foxp3. **D.** The proportion of CD25^+^Foxp3^+^ T cells in the CD4^+^ cell population of each experimental group. **P < 0.01.

To examine whether reduced allostimulatory activity of Mino-DCs was accompanied by an increase in Foxp3^+^ T cells, Ctrl-DCs and Mino-DCs generated from BM cells of C57BL/6 mice were stimulated with LPS for 24 h to mature, and transferred into BALB/c mice. DCs generated from BM cells of BALB/c mice served as a syngeneic DC control. After 7 days, spleen cells were isolated and stained with mAbs specific for mouse CD4, CD25, and Foxp3. Cells were gated on CD4^+^ cells, and then analyzed for the expression of CD25 and Foxp3 (Figure 6A). The proportion of CD25^+^Foxp3^+^ T cells in the CD4^+^ T cell population was significantly increased in Mino-DC-injected mice compared to that in Ctrl-DC-injected mice (7.2% *vs*. 5.4%, *P* < 0.01; Figure 6B), indicating that Mino-DCs caused the induction of Treg cells.

The ability of Mino-DCs to induce the conversion of naïve CD4^+^ T cells to Foxp3^+^ Treg cells was compared with that of Ctrl-DCs *in vitro*. In this experiment, Mino-DCs and Ctrl-DCs generated from BM cells of C57BL/6 mice were pulsed with the OVA323-339 peptide, washed, and then co-cultured in the presence of 100 U/ml IL-2 with CD4^+^ T cells isolated from OT-II mice. After 4 days, cells were harvested, and stained with mAbs specific for mouse CD4, CD25, and Foxp3. Cells were gated on CD4^+^ cells, and then analyzed for the expression of CD25 and Foxp3 (Figure 6C). The proportion of CD25^+^Foxp3^+^ T cells in the CD4^+^ T cell population significantly increased in Mino-DC co-cultures compared to that in Ctrl-DC co-cultures (12.3% *vs*. 10.1%, *P* < 0.01; Figure 6D), indicating that Mino-DCs could induce Treg cells more efficiently than Ctrl-DCs.

### Mino-DCs express high levels of PDL-1

**Figure 7:**
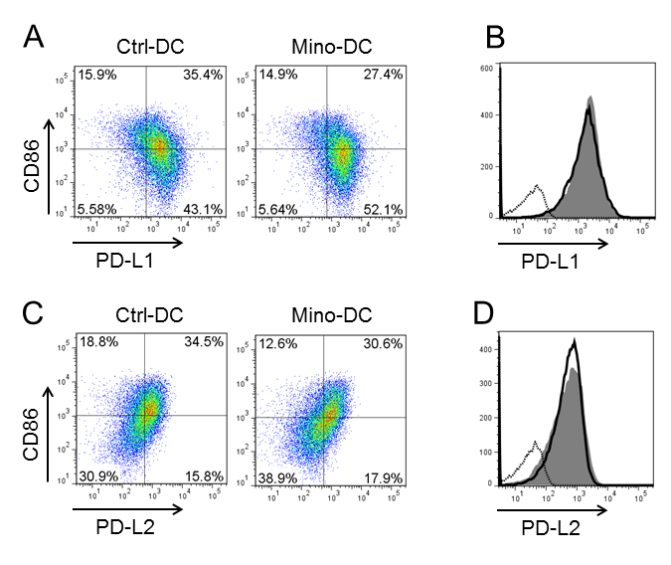
DCs generated in the presence of minocycline express high level of PDL-1 Immature DCs generated from C57BL/6 mouse BM cells with (Mino-DCs) or without (Ctrl-DCs) minocycline were stimulated to maturity with IFN-γ plus TNF-α for 24 h, and analyzed for the expression of PDL-1, PDL-2 and CD86 by flow cytometry. **A.** Representative dot blot histograms showing PDL-1 and CD86 expression. **B.** Mino-DCs, shaded area; Ctrl-DCs, black line; isotype-matched control, dotted line. **C.** Representative dot blot histograms showing PDL-2 and CD86 expression. **D.** Mino-DCs, shaded area; Ctrl-DCs, black line; isotype-matched control, dotted line.

To investigate the mechanism underlying the ability of Mino-DCs to increase the generation of Treg cells, we examined the expression levels of surface PDL-1 and PDL-2 in Mino-DCs. The proportion of PDL-1^hi^CD86^low^ cells was significantly higher in Mino-DCs compared to that in Ctrl-DCs (52.1% *vs*. 43.1%, Figure 7A). In contrast, the proportion of PDL-1^hi^CD86^hi^ cells was much lower in Mino-DCs compared to that in Ctrl-DCs (27.4% *vs*. 35.4%, Figure 7A). Interestingly, no discernible differences could be observed in the overlay histogram (Figure 7B). Unlike PDL-1, there was no significant difference in the proportion of PDL-2^hi^CD86^low^ cells between Mino-DCs and Ctrl-DCs (Figure 7C, 7D). These results confirm the regulatory properties of Mino-DCs.

### Pretreatment with MOG35-55 peptide-pulsed Mino-DCs ameliorates clinical signs of experimental autoimmune encephalitis (EAE)

**Figure 8:**
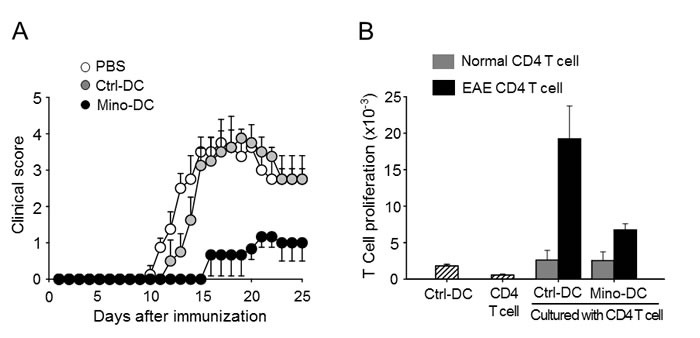
Treatment with MOG peptide-pulsed Mino-DCs ameliorates EAE **A.** Mice were injected i.v. with MOG peptide-pulsed Mino-DCs on days -7 and -3, and EAE was induced on day 0. The clinical score was checked every third day. Three independent experiments were performed, and a representative result is shown; the data are expressed as the mean ± SD for at least four animals. **B.** CD4 T cells were isolated from the spleens of PBS-treated EAE mice (EAE CD4 T cell) or normal mice (Normal CD4 T cell), and then restimulated with MOG peptide-pulsed Ctrl-DCs or Mino-DCs.

The murine EAE model was used to examine the regulatory function of Mino-DCs *in vivo*. Female C57BL/6 mice were injected with MOG35-55 peptide-pulsed Mino-DCs on days -7 and -3, and EAE was induced on day 0. Control mice received MOG peptide-pulsed Ctrl-DCs or PBS on the same days. Pretreatment with MOG peptide-pulsed Mino-DCs significantly reduced EAE (Figure 8A). To examine antigen-specific T cell stimulatory capacity of Mino-DCs, CD4 T cells were isolated from the spleens of PBS-treated EAE mice, and then restimulated with MOG peptide-pulsed Mino-DCs or Ctrl-DCs. The results indicate that MOG peptide-pulsed Mino-DCs had a much weaker ability to induce the proliferation of EAE CD4 T cells (Figure 8B).

## DISCUSSION

The results presented here show, for the first time, that minocycline significantly (by more than 60%) enhanced the generation of DCs from BM cells exposed to GM-CSF and IL-4. The present study also demonstrates that Mino-DCs had the properties of regulatory DCs. Mino-DCs were refractory to LPS- or cytokine (IFN-γ plus TNF-α)-induced maturation, and deficient in MHC class II-restricted exogenous Ag presentation, cytokine production, and allogeneic T cell stimulatory activity. Mino-DCs also induced the expansion of CD4^+^CD25^+^Foxp3^+^ T regulatory cells both *in vitro* and *in vivo*. The therapeutic potential of Mino-DCs was also demonstrated in the EAE model.

Minocycline, a second-generation semi-synthetic tetracycline derivative, has been shown to be beneficial in the treatment of various diseases [29], including autoimmune disorders such as rheumatoid arthritis, inflammatory bowel diseases, scleroderma, aortic aneurysms, and periodontitis [30-32]. Based on the effects minocycline exerts in brain ischemia [33] and neurodegenerative conditions such as Huntington's disease [34], Parkinson's disease [35,36], Alzheimer's disease [37], and multiple sclerosis [38], it is considered the most potent among tetracycline derivatives in regard to neuroprotection [39]. Unlike antimicrobial activity, the anti-inflammatory, immunosuppressive, and neuroprotective properties of minocycline have not been fully understood. The anti-inflammatory and immunosuppressive effects of minocycline are thought to be mediated through inhibition of proinflammatory cytokines such as TNF-α, IL-1β, and IL-6 [40-42], suppression of matrix metalloproteinases [43], inhibition of Ag processing and presentation [44], down-regulation of MHC class II expression [45], and suppression of T cell proliferation and activation [46-48]. Here, we show that the immunosuppressive activity of minocycline is also mediated by the induction of regulatory DCs confirmed both *in vitro* and *in vivo*.

Tolerogenic DCs have attracted significant attention because of their important roles in inducing and maintaining immune tolerance [11]. Tolerogenic DCs are characterized by reduced expression of costimulatory molecules and IL-12 and decreased ability to induce T cell proliferation in parallel with increased IL-10 secretion and Treg stimulation [6-8]. The mechanisms underlying tolerogenic DC activity include the deletion of T cells, induction of Tregs and anergic T cells, expression of immunomodulatory molecules such as PD-L1, and production of immunosuppressive factors such as IL-10, TGF-β, indoleamine 2,3-dioxygenase (IDO), IL-27, and NO [11, 49-51]. In the present study, we found that T cells primed with allogeneic Mino-DCs *in vivo* demonstrated profoundly reduced proliferative capacity in response to re-stimulation with alloantigens (Figure 5B and 5C), and concomitant expansion of CD4^+^CD25^+^Foxp3^+^ Treg cells (Figure 6A). Furthermore, we found that Mino-DCs could induce the conversion of naïve CD4+ T cells to Treg cells (Figure 6C). These results suggest that the increase in Tregs may be a mechanism underlying the regulatory properties of Mino-DCs. However, further work is required to elucidate the full tolerogenic potential of Mino-DCs.

Numerous pharmacological agents have been shown to induce regulatory DCs, including vitamin D3, dexamethasone, rapamycin, corticosteroid, aspirin, atorvastatin, retinoic acid, and mycophenolic acid [7-8, 15-23]. In this context, the most important feature of minocycline is a significant increase in the number of regulatory DCs generated from BM cells stimulated with GM-CSF and IL-4. Rapamycin used to produce tolerogenic DCs has been reported to significantly reduce the number of BM-derived CD11c^+^ cells [6]. Dexamethasone has also been shown to markedly affect cell recovery [18,19]. In contrast, minocycline significantly (by more than 60%) increased the number of BM-generated DCs (Figure 1).

The effects of minocycline on the generation of regulatory DCs from BM cells appear to be different from those on immature DCs differentiated for 6 days in the presence of GM-CSF and IL-4. To examine whether minocycline could render immature DCs regulatory, DCs generated in minocycline-free conditions were exposed to minocycline with or without maturation stimuli (IFN-γ plus TNF-α). Minocycline did not affect DC maturation when added to immature DCs together with maturation agents, indicating that DCs with regulatory properties were obtained when BM cells were exposed to minocycline during DC development. However, the underlying mechanisms are currently unknown.

In conclusion, our data suggest that minocycline can be used to enhance the production of regulatory DCs from BM cells induced with GM-CSF and IL-4. The advantage of minocycline is its safety in chronic application, and a long half-life in the body. Thus, our present study revealed a novel pharmacological agent that can be used in generating regulatory DCs *in vitro* and *in vivo* for possible use in treating autoimmune disorders and allergies.

## MATERIALS AND METHODS

### Mice

Female C57BL/6 and BALB/c 8-12-week-old mice were purchased from Orient Co. Ltd. (Seoul, Korea). All animal protocols were approved by the Animal Care Committee of Chungbuk National University, Cheongju, South Korea.

### Generation of DCs from BM cells

DC_S_ were generated as described previously [52]. Briefly, total BM cells obtained from mouse femurs were cultured in 6-well plates (5 × 10^6^/well) in culture medium containing 20 or 40 ng/ml GM-CSF and 20 or 40 ng/ml IL-4 (both from Creagene, Seongnam, Korea). To generate Mino-DCs, 5 μM minocycline (Santa Cruz Biotechnology, Santa Cruz, CA, USA) was added at the start of the culture. After 3, 4, and 6 days, non-adherent cells were removed by gentle shaking and replacing the medium. DCs were harvested by gentle pipetting on day 7. In some experiments, the harvested DCs were exposed to 100 ng/ml LPS (Sigma-Aldrich, St. Louis, MS, USA) or 50 ng/mL IFN-γ plus 50 ng/mL TNF-α (PeproTech, Rocky Hill, NJ, USA) for 24 h to induce maturation.

### Preparation of microspheres

Microspheres containing OVA (OVA-microspheres) were prepared using a homogenization/solvent evaporation method described previously [53]. Briefly, 400 ml of 50 mg/ml OVA in water was mixed with 2 ml of 100 mg/ml poly(lactic-co-glycolic acid) in ethyl acetate (Sigma-Aldrich). The OVA content was determined using the Micro Bicinchoninic Acid assay kit (Pierce, Rockford, IL, USA) after lysing the microspheres in lysis buffer (0.1 % SDS and 0.1 N NaOH). Fluorescence-labeled microspheres were prepared by adding FITC (5 mg/ml) to the ethyl acetate phase.

### MHC class II-restricted Ag presentation assay

The class II-restricted exogenous Ag presentation assay was performed as described previously using OVA-specific CD4 T cell hybridoma DOBW cells which recognize OVA [323-339]-I-A^d^ complexes and secrete IL-2 in response [54]. Briefly, DCs seeded into 96-well flat bottom plates (1 × 10^5^/well) were mixed with the indicated amounts of OVA-microspheres. After 1-h incubation at 37°C, cells were washed twice with 300 ml/well of pre-warmed PBS, and fixed with 100 ml/well of pre-warmed 1 % paraformaldehyde for 5 min at room temperature. Following three washes with 300 ml/well PBS, DOBW cells were added (2 × 10^5^/well) for 18 h at 37°C. Plates were centrifuged at 400 × *g*, and culture supernatants were collected and assayed for IL-2 levels using the IL-2 ELISA kit (BD Biosciences, San Jose, CA, USA).

### Phagocytic activity

FITC-labeled OVA-microspheres (1 mg/well OVA) were added to DCs for 2 h; non-phagocytosed microspheres were removed by washing twice with pre-warmed PBS. Cells were harvested, fixed in 1% paraformaldehyde in PBS, and analyzed by flow cytometry (FACS Calibur, Becton Dickinson, Franklin Lakes, NJ, USA).

### Preparation of T cells

Total T cells were purified from the spleen of BALB/c mice by adding the spleen cells to a nylon wool column and incubating for 1 h to remove adherent cells. CD4^+^ T cells were isolated from the adherent cell-depleted spleen cell population using the CD4^+^ isolation kit (Militeny Biotec, Bergisch Gladbach, Germany).

### Mixed lymphocyte reaction

To assay lymphocyte proliferation in the mixed lymphocyte reaction, allogeneic CD4^+^ T cells seeded in 96-well round bottomed plates (1 × 10^5^/well) were stimulated with different concentrations of DCs for 72 h. DNA synthesis was measured by the incorporation of [^3^H]-thymidine (0.5 mCi/well; Du Pont, Boston, MA, USA) added for the final 18 h of culture.

### Generation of Mino-DCs *in vivo*

C57BL/6 mice were given i.p. injections of GM-CSF (2 μg/mouse, twice daily) and minocycline (10 mg/kg, twice daily) for 6 consecutive days; control mice received PBS. On day 7, mice were sacrificed, and the spleens were removed and used to obtain single-cell suspensions by teasing and passing through a 70-μm cell strainer (BD Falcon, San Jose, CA, USA). Erythrocytes were lysed by adding ACK lysis buffer (0.15 M NH_4_Cl, 1.0 mM KHCO_3_, 0.1 mM EDTA) for 3 min, and CD11c^+^ DCs were isolated by negative selection using immunomagnetic beads (MACS, Miltenyi Biotec Inc., Auburn, CA, USA). The purity of the selected CD11c^+^ DC population was > 90 %, as assessed by flow cytometry.

### *In vitro* re-challenge experiments

CD11c^+^ DCs isolated from C56BL/6 mice treated with GM-CSF alone or GM-CSF plus minocycline were transferred i.v. to BALB/c mice. Ten days later, T cells were isolated from the immunized mice using the Pan T cell isolation kit (MACS, Miltenyi Biotec Inc., Auburn, CA, USA) and cultured in 96-well plates (2 × 10^5^/well) with different concentrations of irradiated normal DCs generated from BM cells of naive C57BL/6 mouse. T cell proliferation was measured after 3 days by [^3^H]-thymidine incorporation as described above.

### *In vitro* generation of CD4^+^ Treg cells from naïve CD4^+^ T cells

Naïve CD4^+^ T cells were isolated from the spleens of OT-II mice using a CD4^+^ T cell isolation kit (Miltenyi Botec Inc.). Both Mino-DCs and Ctrl-DCs were generated from BM cells of C57BL/6 mice, matured in the presence of 50 ng/mL IFN-γ and 50 ng/mL TNF-α for 24 h, and then pulsed with the OVA323-339 peptide (10 μg/ml) for 1 h. After washing with PBS, DCs (1 × 10^4^ cells/well) were co-cultured with purified OT-II CD4^+^ T cells (2 × 10^5^ cells/well) for 4 days in the medium containing 100 U/ml of recombinant human IL-2 (PeproTech Inc., Rocky Hill, NJ, USA).

### Flow cytometry analysis

Cells were stained as described previously [52] with monoclonal antibodies against mouse cell surface markers I-A^b^, CD80, CD86, CD54, CD11c, CD4, CD25, and Foxp3, and an isotype-matched control antibody (BD Biosciences). For intracellular Foxp3 staining, cells were permeabilized using the BD Cytofix/Cytoperm Plus kit according to the manufacturer's instructions (BD Biosciences). Subsequent analysis was performed using the FlowJo software (TreeStar, Ashland, OR, USA).

### Induction of EAE and pretreatment with MOG35-55 peptide-pulsed DCs

Immature Mino-DCs and Ctrl-DCs were induced to maturation on day 6 by exposure to 50 ng/mL IFN-γ plus 50 ng/mL TNF-α for 24 h. Both cells were then pulsed with 25 mg/mL MOG35-55 peptide for 1 h at 37°C, harvested by gentle pipetting, washed, and injected into female C57BL/6 mice (1.2 × 10^6^/mouse, i.v.) on days -7 and -3; then EAE was induced on day 0. On the day of EAE induction, mice were immunized s.c. with 100 mg of the MOG35-55 peptide (Peptron, Daejeon, Korea) in 100 ml of PBS emulsified in 100 ml of complete Freund's adjuvant (Sigma-Aldrich) containing 4 mg/mL *Mycobacterium tuberculosis* (H37Ra, Difco/BD Bioscience). In addition 200 ng of pertussis toxin (Sigma-Aldrich) was injected i.p. on days 0 and 2. EAE paralysis in mice was scored as follows: 0, no symptoms; 1, flaccid tail; 2, hind limb weakness; 3, partial hind limb paralysis; 4, complete hind limb paralysis; 5, moribund state (55).

### Statistical analysis

Student's t test was performed for a single comparison of two groups after evaluation for normality. A Man-Whitney rank sum test was performed if data distribution was not normal. One- or two-way analysis of variance was applied to compare multiple groups; *p* < 0.05 was considered statistically significant.

## SUPPLEMENTARY MATERIAL FIGURE


