# Supplementary material for: Minocycline promotes the generation of dendritic cells with regulatory properties
Source: Oncotarget. 2016 Jul 24;7(33):52818–31. doi: 10.18632/oncotarget.10810 (PMC5288151; doi:10.18632/oncotarget.10810)
Supplement: Supplementary file 1 [file oncotarget-07-52818-s001.pdf]

Minocycline promotes the generation of dendritic cells with regulatory properties

Supplementary Material

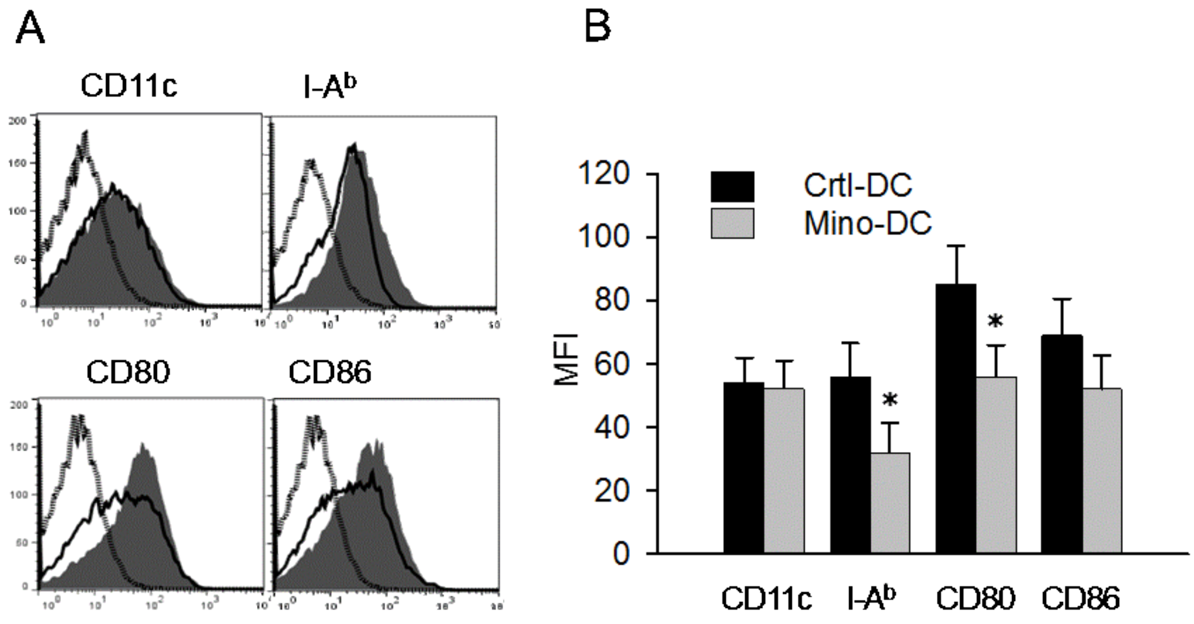

Supplementary Figure S1. Mino-DCs isolated from the spleens of mice treated with GM-CSF plus minocycline express significantly lower levels of I-A^b and CD80 compared to Ctrl-DCs isolated from the spleens of mice treated with GM-CSF alone. (A) Ctrl-DCs, shaded area; Mino-DCs, black line; isotype-matched control, grey line. (B) Mean fluorescence intensities of DCs. The data are presented as the mean ± SD of three independent experiments; *P < 0.05 compared with control.
